# Epidemiological exploration of fleas and molecular identification of flea-borne viruses in Egyptian small ruminants

**DOI:** 10.1038/s41598-024-64881-0

**Published:** 2024-07-02

**Authors:** Safaa M. Barghash, Samah E. Yassin, Al-Shaimaa M. Sadek, Dalia M. Mahmoud, Mohamed S. Salama

**Affiliations:** 1 Parasitology Unit, Animal Production and Poultry Division, Animal and Poultry Health Department, Desert Research Center, El-Naam, Cairo, Egypt; 2 Zoology and Entomology Department, Faculty of Science, Al-Azhar University, Nasr City, Cairo, Egypt; 3 Department of Entomology, Faculty of Science, Ain Shams University, Cairo, Egypt

**Keywords:** Fleas, Lumpy skin disease, Coronavirus, Capripoxvirus, Genotyping, Egypt, Ecology, Microbiology, Molecular biology, Zoology

## Abstract

The study aimed to investigate molecularly the presence of flea-borne viruses in infested small ruminants with fleas. It was carried out in Egypt’s Northern West Coast (NWC) and South Sinai Governorate (SSG). Three specific primers were used targeting genes, ORF103 (for Capripoxvirus and Lumpy skin disease virus), NS3 (for Bluetongue virus), and Rdrp (for Coronavirus), followed by gene sequencing and phylogenetic analyses. The results revealed that 78.94% of sheep and 65.63% of goats were infested in the NWC area, whereas 49.76% of sheep and 77.8% of goats were infested in the SSG region. Sheep were preferable hosts for flea infestations (58.9%) to goats (41.1%) in the two studied areas. Sex and age of the animals had no effects on the infestation rate (p > 0.05). The season and site of infestation on animals were significantly different between the two areas (p < 0.05). *Ctenocephalides felis* predominated in NWC and *Ctenocephalides canis* in SSG, and males of both flea species were more prevalent than females. Molecular analysis of flea DNA revealed the presence of Capripoxvirus in all tested samples, while other viral infections were absent. Gene sequencing identified three isolates as sheeppox viruses, and one as goatpox virus. The findings suggest that Capripoxvirus is adapted to fleas and may be transmitted to animals through infestation. This underscores the need for ongoing surveillance of other pathogens in different regions of Egypt.

## Introduction

The genus Capripoxvirus (CaPVs), has three species of poxvirus: goatpox virus (GTPV), sheeppox virus (SPPV), and lumpy skin disease virus (LSDV)^1^. These viruses cause serious transboundary illnesses that can seriously harm livestock production systems. The Capripox virus, a part of the Poxviridae family, is a major global threat to the health and production of sheep and goats^2^. This virus is responsible for the highly transmissible condition known as Capripox, characterized by a severe febrile illness accompanied by the formation of nodules on the skin, lesions on mucous membranes, and extensive lymphadenopathy^3^. Beyond the obvious clinical symptoms, a CaPV infection can have a significant negative economic impact due to decreased milk supply, weight loss, reduced fertility, and high mortality rates, especially in young animals^4^. Furthermore, the CaPV virus poses a greater threat to small ruminant farming communities because of its capacity to spread quickly within susceptible populations. Close contact and mechanical transmission aided by arthropod vectors such as ticks, mosquitoes, and fleas^5^. Understanding the impact of the CaPVs on sheep and goats is crucial for implementing preventive strategies and reducing the socio-economic burden caused by this devastating disease. There are significant geographic distributions for CaPV infections, in most of Africa, the Middle East, Central Asia, and the Indian subcontinent, SPPV and GTPV are endemic^6^. LSDV, on the other hand, is mostly found in southern, central, eastern, and western Africa; its presence in the north Sahara Desert and outside of Africa was initially documented in Egypt in 1988 and 1989, and it was then reported in 2006, 2011, and 2014^7^.

The bluetongue (BTV) is a non-contagious, insect-borne infection that affects ruminants. It is a member of the Orbivirus genus within the Reoviridae family^8^. The infection is found in Africa, Europe, the Middle East, and the Mediterranean region^9^. Both domestic and wild ruminants are susceptible to being infected with bluetongue, although cattle and wild ruminants serve as significant reservoir hosts for the virus, sheep and goats are the primary hosts of the disease^10^. The World Organization for Animal Health (OIE) emphasizes the importance of maintaining essential veterinary regulatory services, food safety inspection, emergency response measures, and potential vectors that may transmit it, vaccination programs against diseases affecting public health and the economy, and prioritized research activities during the COVID-19 pandemic^11^. Villar et al.^12^ suggested that cat fleas may serve as biological and/or mechanical vectors of coronaviruses, prompting the need for further studies to confirm the role of animal hosts and their ectoparasite vectors in the transmission of coronavirus. This study contributes to these efforts by investigating the potential transmission of coronaviruses by fleas to small ruminants in the surveyed areas.

Fleas are highly specialized blood-sucking ectoparasites. Several diseases of medical and veterinary importance, including bacteria, viruses, and parasites, can be spread by fleas in a variety of ways, including through bites (both biological and mechanical), faeces, regurgitation, and ingestion of infected fleas by the host^13^. Today, it is possible to discriminate between approximately 15 families, 220 genera, and 2500 flea species^14^. Only five families and 25 genera of fleas are ectoparasites of birds; all other fleas parasitize mammals^15^. Most fleas of veterinary importance are classified in the families Pulicidae, Ceratophyllidae, Leptopsyllidae, and Vermipsyllidae^16^. Domestic cats and dogs play essential or accidental roles in the natural transmission cycle of flea-borne pathogens^17^. They either support the growth of some pathogens or serve as transport vehicles for infected fleas between their natural reservoirs and hosts^18^.

Recent decades have seen significant shifts in vector-borne infections and diseases, with global changes such as climate and land use influencing the distribution and density of hematophagous arthropod species^19^. Genetic techniques can confirm species, but not all strains. Advancements in molecular biological techniques allow prompt and accurate diagnosis of viral infections^20^. However, duplication, gene transfer, low specificity, and gene loss can affect the reliability of results^21^. Researchers at Desert Research Center stations in Egypt’s northern West Coast and South Sinai Governorates have reported a significant increase in the flea epidemic. Despite attempts to resolve the issue, the situation worsens during spring and summer due to the large number of animals, birds, and humans, and the overcapacity for replacement. The current study aimed to address those possible flea-borne viruses in these stations and their territorial scope, starting with the identification of flea-infested ruminants in Egypt’s Northern West Coast (NWC) and South Sinai Governorate (SSG).

## Materials and methods

### Ethics statement

The study was approved by the ethics committee at the Desert Research Center (No. ACUC-APPD-APPPD-DRC-#39#), which is compatible with the International Animal Ethics Committee (8th Edition 2011, Record number: 13799, legacy ID: 8247) and according to the ARRIVE guidelines.

### Study area

**Figure 1:**
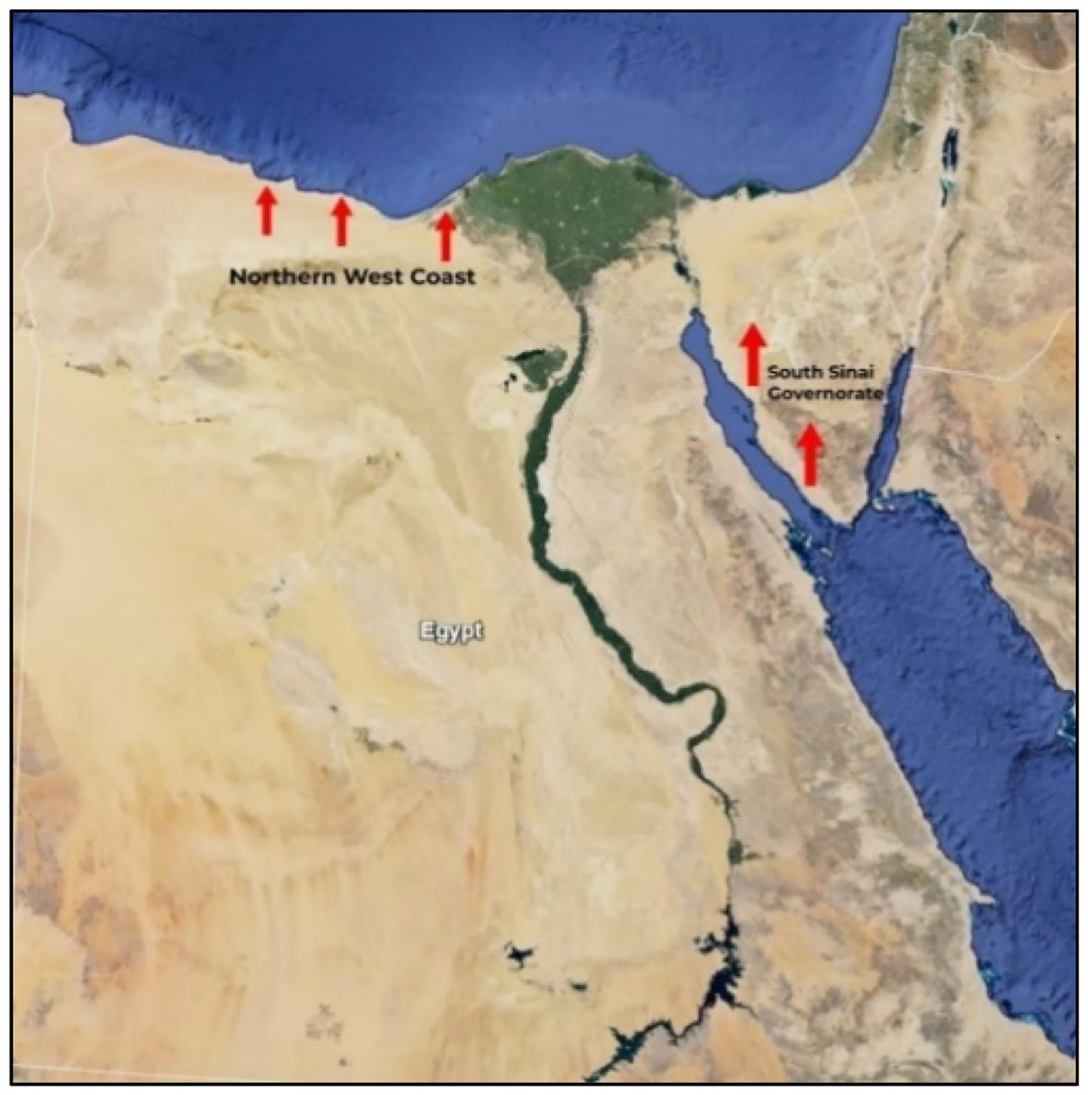
Google Map of Egypt shows the surveyed areas where samples were collected.

The study was conducted in NWC and SSG from April 2020 to September 2021. The coordinates are 29° 50′ 00′′ E and 25° 10′ 00′′ E, and the latitudes are 30° 50′ 00′′ N and 31° 10′ 00′′ N for the NWC and 29° 03′ N 33° 50′ E and 29.05° N 33.83° E for the SSG (Fig. 1). The climates of the two regions vary widely. Most of the NWC area enjoys a healthy, moderate Mediterranean climate, unlike the SSG area, which has a desert climate for most of the year. Arthropod vectors, such as ticks and fleas, increase in the dry season in both study regions. There are more Barki sheep, which are dominant in the NWC, whereas Baladi and Shami goats are predominant in the SSG.

### Animals and flea collection

To identify flea infestations, a total of 765 randomly selected animals were examined, comprising 460 sheep and 305 goats from both regions. Among them, 390 sheep and 265 goats were female, while 70 sheep and 40 goats were male, with ages ranging from 9 months to 5 years across both areas. Flea species were collected from infested small ruminants in the NWC and SSG (each region individually) using flea combs on wool after using an anaesthetic spray (Lidocaine Spray) for a short time. Flea collection was something difficult for sheep but easier for goats. Specimens were preserved in tubes containing 70% ethyl alcohol. The collected fleas were then subdivided into pools, each containing a maximum of 75 fleas of the same species, sex originating from both locations.

### Microscopical identification of fleas

Fleas were identified and classified into species using Rothschild^22^ methodology, employing an Olympus SZ-PT-40 microscope. Subsequently, the fleas were segregated into pooled samples consisting of all sexes and belonging to the same species.

### Molecular studies of flea-borne viruses

#### Oligonucleotide primers and cycling conditions

Based on previous studies that reported their potential, oligonucleotide primers with specific sequences supplied by Metabion (Germany) were employed. They amplified specific products of different sizes, as shown in Table 1.

**Table 1:** Oligonucleotide primer sequences for viruses.

Agent (gene)	Sequence	Bp	Ref.
Capripox and Lumpy skin diseases (ORF103)	ATGTCTGATAAAAAATTATCTCG	570	Zhao et al.^23^
	ATCCATACCATCGTCGATAG		
Bluetongue (NS3)	TCGCTGCCATGCTATCCG	251	Akita et al.^24^
	CGTACGATGCGAATGCAG		
Coronavirus (Rdrp)	CCAARTTYTAYGGHGGITGG	670–673	Xiu et al.^25^
	TGTTGIGARCARAAYTCATGIGG		

#### DNA extraction and analysis protocol for flea samples

Following the protocol and guidelines outlined in the blood and tissue DNeasy mini kit (Qiagen, Hilden, Germany), DNA extraction was performed. Pooled flea samples were homogenized in 180 μl of ATL (tissue lysis buffer) using a tissue Lyzer (Qiagen, Hilden, Germany) equipped with a 5 mm steel bead. Subsequently, the recovery and purity of each DNA sample were assessed using a spectrophotometer (NanoDrop® ND-1000, PeqLab, Erlangen, Germany). The extracted DNA was then stored at − 20 °C until further use.

#### PCR amplification

Each reaction included 12.5 μl of Emerald Amp GT PCR master mix (2× premix), 5 μl of template DNA, 1 μl each of forward and reverse primers, and 5.5 μl of PCR-grade water in a total volume of 25 μl. The researchers followed the instructions in the QuantiTect probe RT‒PCR kit handbook (2008) for RNA agents to carry out the reaction. DNA amplification started with a reverse transcription step for only the Bluetongue and Corona viruses, not the Capripox viruses, followed by one cycle of pre-denaturation at 95 °C for 5 min and 35 cycles of secondary denaturation at 94 °C for 30 s. The annealing temperatures were 52 °C for 30 s (Bluetongue), 53.4 °C for 40 s (Corona), and 52 °C for 40 s (Capripox and Lumby skin diseases), and the extension step was 52 °C for 30 s. Finally, we performed one cycle of post- extension at 72 °C for 10 min.

### Molecular characterization of flea-borne viruses

#### Purification of PCR products

The QIAquick PCR Product Extraction Kit (Qiagen Inc., Valencia, CA) was used for the purification of each PCR product directly, and the purified PCR products were sequenced in the forward and reverse directions on an Applied Biosystems 3130 automated DNA sequencer (ABI, 3130, USA).

#### Gene sequencing and phylogenetic analyses

BLAST® analysis (Basic Local Alignment Search Tool) Altschul et al.^26^ was used to determine the sequence identity of the GenBank accessions. The sequences were compared to those of closely related preserved species in the GenBank database using the ClustalW algorithm according to Thompson et al.^27^. The neighbour-joining model was used to construct phylogenetic trees, and similarities between isolates were determined using the maximum likelihood test in Molecular Evolutionary Genetics Analysis Version 6.0 (MEGA6) software^28^. The phylogenetic robustness was assessed by undertaking bootstrap re-sampling using 1000 replicates.

### Statistical analysis

The data was analysed using least squares analysis of variance with the general linear model procedure^29^. A Chi-squared test was used to analyse categorical data. All statements of significance are based on a probability of less than 0.05.

## Results

### Prevalence and distribution of fleas

**Figure 2:**
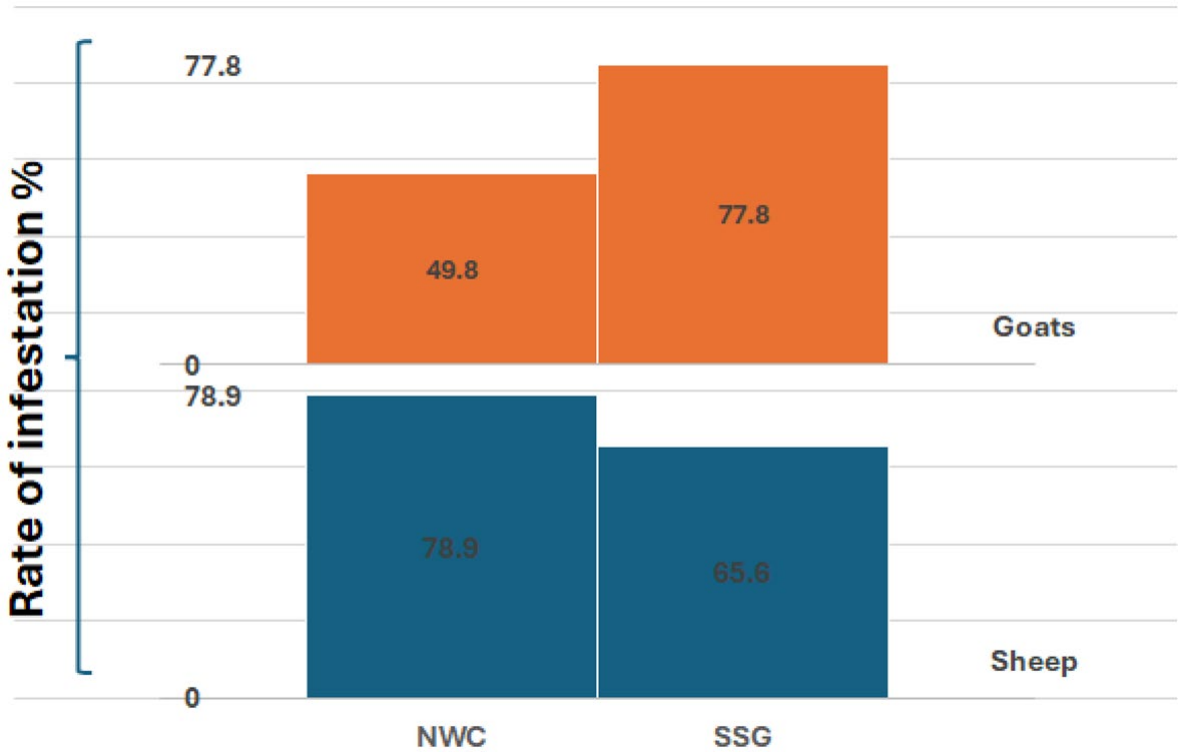
A descriptive comparison chart, created using the multi-layer column chart feature in the KUTOOLS program for Excel, illustrates the rate of flea infestation (%) among animals in two investigated sites: the Northern West Coast (NWC) and the South Sinai Governorate (SSG).

**Figure 3:**
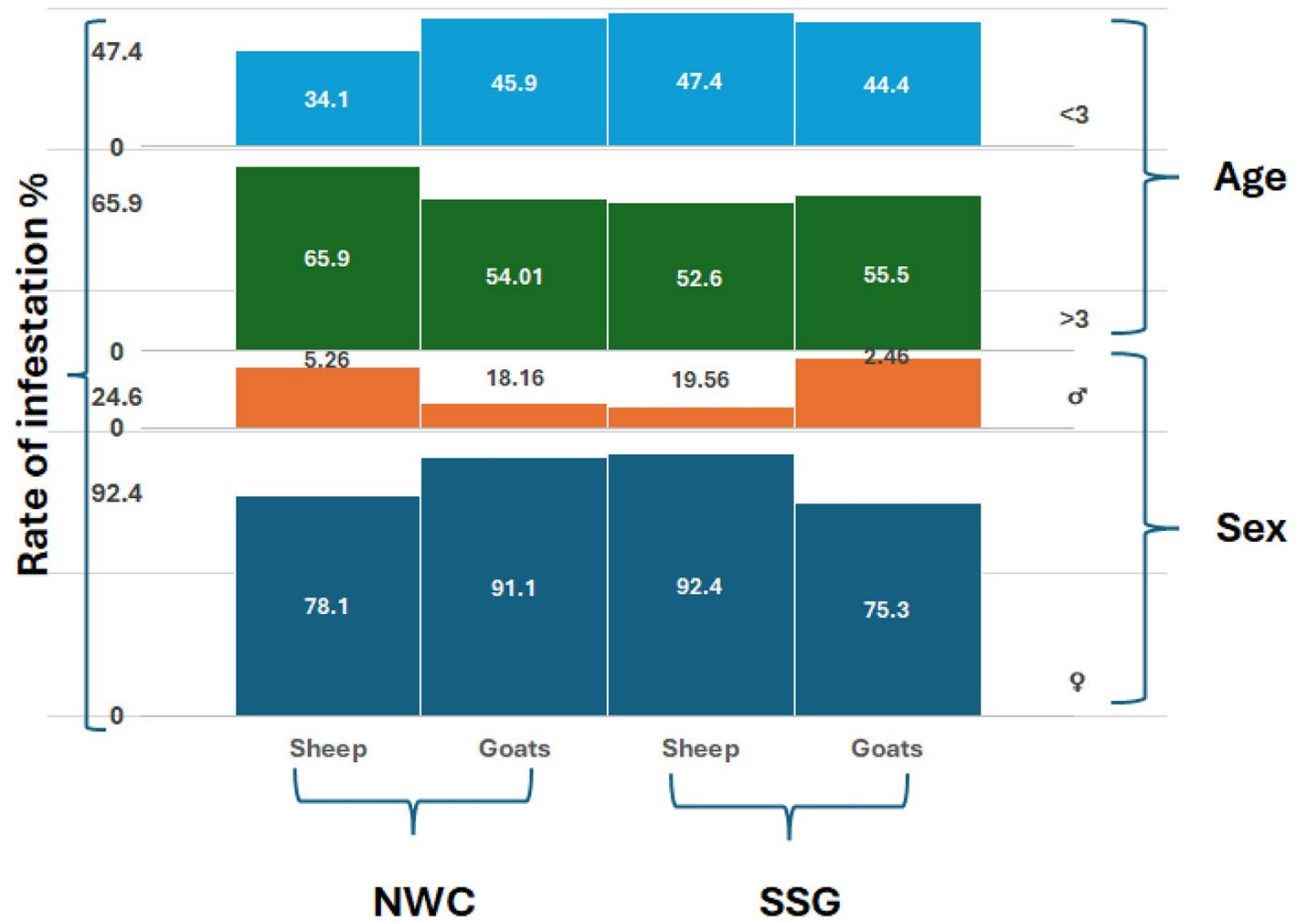
A descriptive comparison chart, created using the multi-layer column chart feature in the KUTOOLS program for Excel, illustrates the rate of flea infestation (%) among animals according to age and sex in two investigated sites: the Northern West Coast (NWC) and the South Sinai Governorate (SSG).

Of the 511 infested animals, 434 (66.3%) females and 77 (70.0%) males harboured fleas. These percentages were 78.9% and 65.6% in sheep and goats, respectively, in the NWC area and 49.8% in sheep and 77.8% in goats in the SSG area (Fig. 2). The rates were relatively similar for female and male animals in the two areas. Whereas female sheep had the highest infestation rate in the NWC and the lowest rate in the SSG, male goats had the highest infestation values compared to male sheep in the two areas. Concerning the age of the examined animals, older animals had greater infestation rates than younger animals in the NWC area. The prevalence of flea-infested ruminants according to the sex and age of the host showed no significant differences between the ages of the infested animals with fleas in the two study areas, except for that observed between the two aged groups of sheep in the NWC area (p < 0.01). However, for animals older than three years, infestation rates of 81.6% and 79.3% were recorded for sheep and goats, respectively. For animals younger than three years, infestation rates of 73.8% and 49.5% were recorded for sheep and goats, respectively. Older sheep in the SSG area had the lowest infestation rate of 31.25%, whereas older goats had the maximum infestation rate of 80%. The younger-aged animals exhibited relatively similar infestation rates, ranging from 70.3 to 75% (Fig. 3).

**Figure 4:**
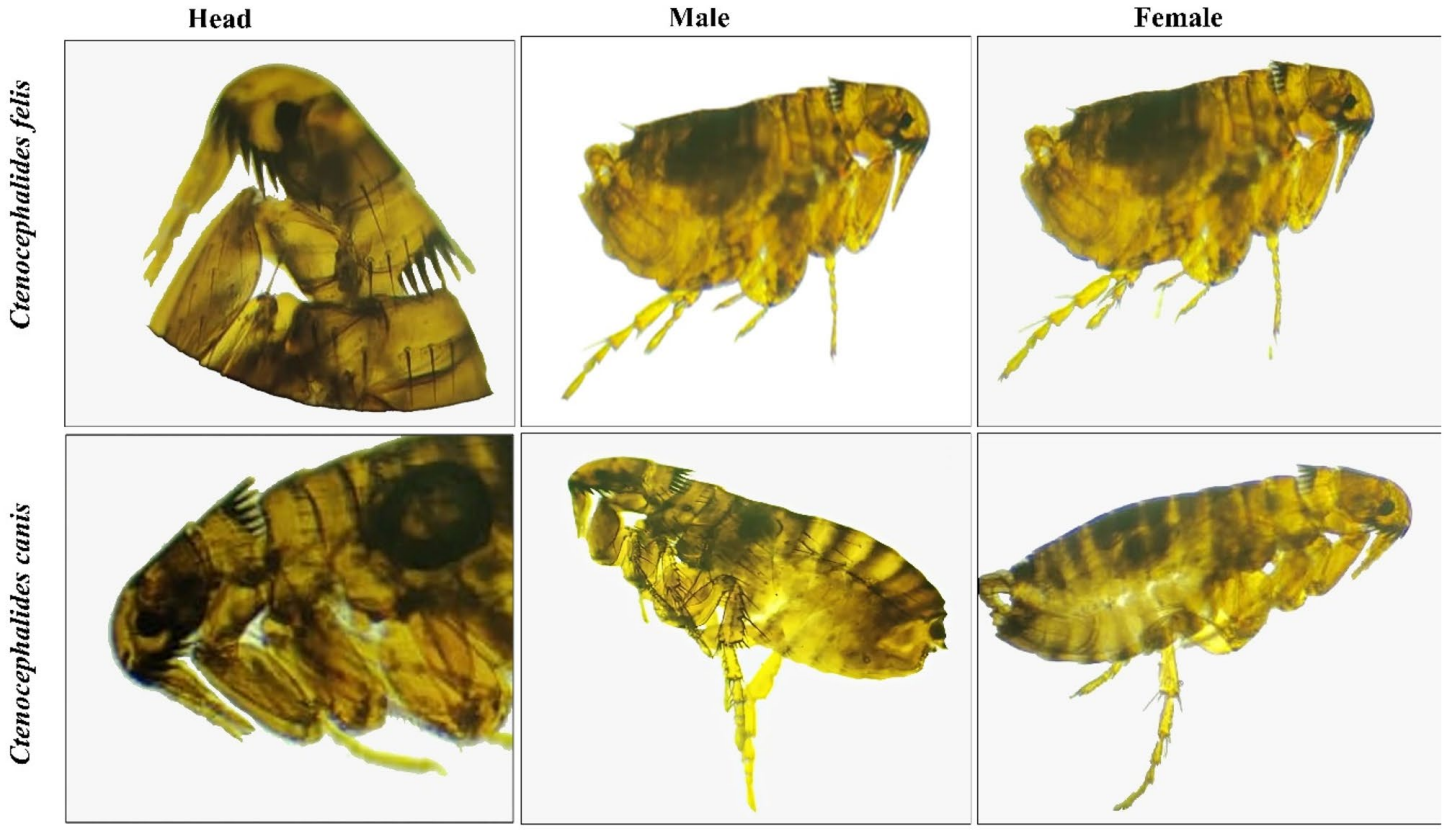
Shows the microscopical identification of fleas sp. during the study.

**Figure 5:**
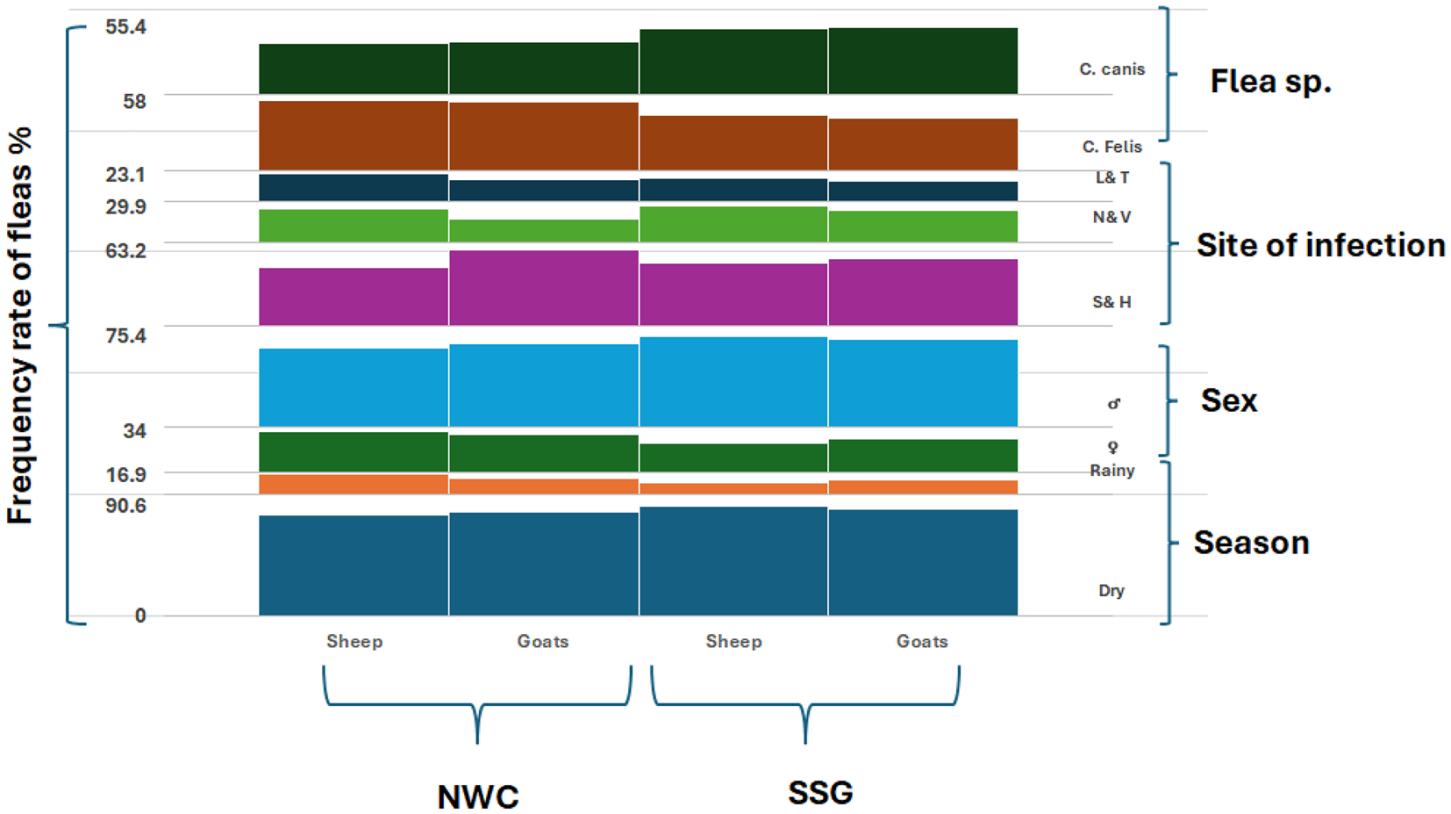
A descriptive comparison chart, created using the multi-layer column chart feature in the KUTOOLS program for Excel, illustrates the frequency rate of the fleas (%) collected during the study according to the species of fleas, site of infection (*S&H* Sexual organs and Head, *N&V* Neck and Ventral, *L&T* Legs and Tail), sex of the fleas and the season of collection in two investigated sites: the Northern West Coast (NWC) and the South Sinai Governorate (SSG).

*Ctenocephalides* was the only one collected from sheep and goats from the two different localities (Fig. 4). The present study revealed the presence of fleas on animals throughout the year. These parameters increased more significantly (p = 0.0001) in the dry seasons than in the rainy seasons in the two study areas. The host species and location did not significantly differ among the infestation rates (p > 0.5), and the number of infestation sites on the animal was significantly greater on the head and around sexual organs (54.6%) than that at other sites, such as the neck and ventral region (25.7%) and the legs and tail region (19.7%). Sheep were preferable hosts for flea infestations (58.9%) to goats (41.1%) in the two studied areas, with no significant difference (p > 0.05). There was a statistically significant difference (p = 0.00024) in the species of fleas between the infestations of *C. felis* and *C. canis* in the two regions. In general, 52.8% of the afflicted animals had more cat fleas (*C. felis*) than did 47.18% of the dog fleas (*C. canis*). In the NWC region, *C. felis* predominated at 57.7%, while *C. canis* predominated at 42.3%. Conversely, in the SSG area, *C. canis* was more frequent (54.4%) than *C. felis* (45.6%). In the two locations, males of the two flea species were more common than females. While there were significant differences between the sexes of the two flea species in the SSG (P = 0.79), there were differences between the sexes of the fleas in the NWC (p = 0.004) (Fig. 5).

### PCR and sequence analysis

**Figure 6:**
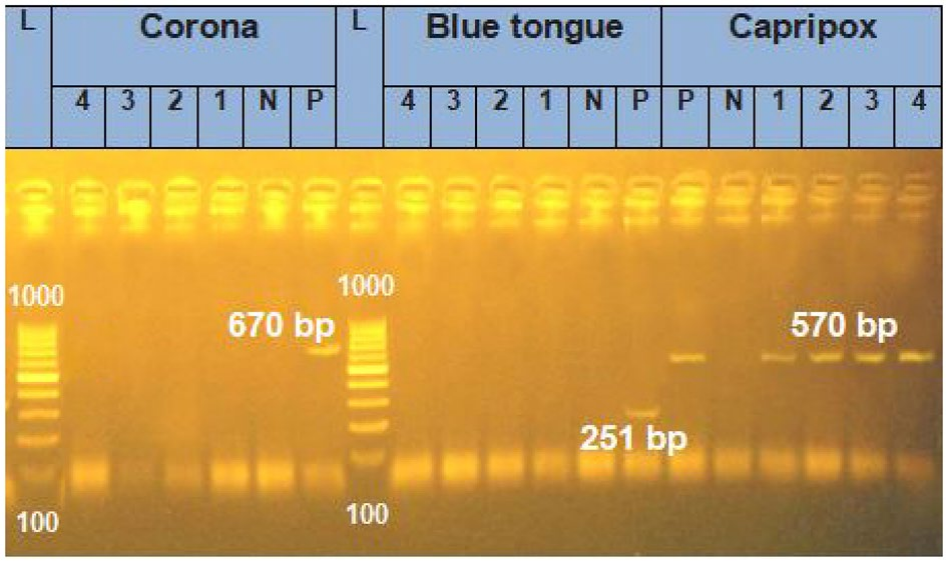
PCR-based assays targeting the ORF103, NS3, and Rdrp genes for detecting Capripox*,* Bluetongue, and Coronaviruses, respectively. L: 100-bp DNA ladder; lanes 1, 2, 3, and 4 correspond to the examined flea-DNA samples. Lanes P and N represent positive and negative controls, respectively.

PCR was employed to confirm the infestation of the collected fleas by viruses. Capripoxvirus was identified in all four examined samples, which encompassed both sexes of the two flea species, yielding an amplicon size of 570 bp. However, the bluetongue, coronavirus, and lumpy skin disease viruses were not detected, as evidenced by the absence of bands at 670 bp and 251 bp, respectively (Fig. 6). Among the four positive results, three were molecularly identified as SPPV and were deposited in GenBank with accession numbers ON081029, ON081030, and ON081031, while the fourth was identified as GTPV with accession number ON081032.

**Figure 7:**
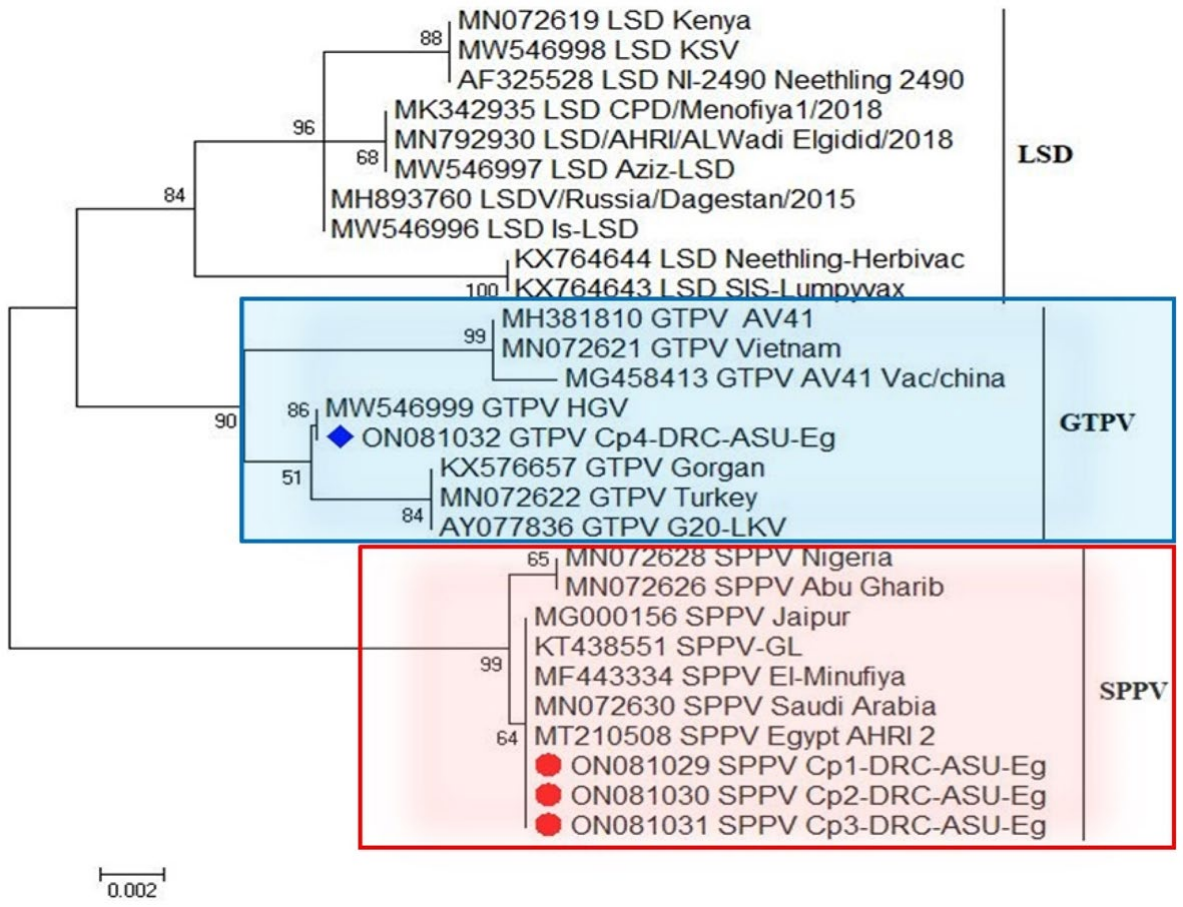
A phylogenetic tree was constructed in MEGA 6.0 using the neighbor-joining method for the analysis of the ORF103 gene for sheep and goat Capripox viruses. The accession numbers obtained in this study are colored.

The nucleotide sequences of sheep and goatpox viruses obtained during this study exhibited high identity with most sequences in GenBank (Table 2). Specifically, the Capripox virus from sheep clustered within the same phylogenetic clade as Capripox viruses isolated from sheep in Egypt, Saudi Arabia, China, and India (Fig. 7), displaying 100% nucleotide sequence identity with all sequenced isolates from these regions (Table 2). While the goatpox virus fell within the same phylogenetic clade as Capripox viruses from Egypt, Iran, Turkey, and Kazakhstan (Fig. 7). The isolated virus exhibited 99.6% similarity with sequences from Iran (KX576657), Turkey (MN072622), and Kazakhstan (AY077836), and the lowest nucleotide sequence identity of 98.4% with a sequence from China (MG458413) (Table 2).

**Table 2:** Nucleotide sequence identity of Capripoxvirus isolates based on the ORF103 gene during this study (ON081029, ON081030, ON081031 [sheeppox virus], and ON081032 [goatpox virus]) with the gene from different countries.

Origin	Acc. No	Country	Identity %	Origin	Acc. No	Country	Identity %
Sheeppox virus (SPPV)	MN072628	Nigeria	100	Goatpox virus (GTPV)	MH381810	China	98.6
	MN072626	Iraq	100		MN072621	Vietnam	98.6
	MG000156	India	100		MG458413	China	98.4
	KT438551	China	100		MW546999	Egypt	100
	MF443334	Egypt	100		KX576657	Iran	99.6
	MN072630	Saudi Arabia	100		MN072622	Turkey	99.6
	MT210508	Egypt	100		AY077836	Kazakhstan	99.6

## Discussion

Studying flea infestation in small ruminants is crucial for assessing health risks, evaluating control measures, and implementing targeted interventions to improve animal welfare and mitigate economic losses. The cat flea *C. felis* and the dog flea *C. canis* are prevalent worldwide and of particular significance for companion animals. *C. felis* has been found on over fifty different host species, which helps to explain its persistence in the environment. In the present study, the increasing threat posed by diseases was reflected in the greater number of fleas on animals in dry seasons than in rainy seasons and certain places on animals. These higher infestation rates were caused by several factors, such as favourable climate conditions, starvation, particularly during dry seasons, ineffective husbandry practices, a lack of knowledge among farmers about the effects of ectoparasites, and a lack of adequate animal health services in the study areas^30^. The results showed that the head and the area around the sexual organs are more susceptible to infestation compared to other parts of the body. This finding is consistent with the research of Roy et al.^31^, who reported that as the number of generations per year increases, the potential density of fleas also increases significantly, particularly around the head, neck, and genital organs rather than other areas on animals. The examination of flea distribution across the two surveyed regions indicated infestations in sheep and goats, with sheep displaying a higher infestation rate than goats. This discrepancy is attributed to the innate self-grooming behaviours of goats, such as licking, scratching, rubbing, and utilizing other self-defence mechanisms^32^. The infestation rate was higher in older animals compared to younger ones, and this difference may reflect the overall health conditions of infested animals^33^ in the present study. Our results contrast with those of Constable et al.^34^, who reported the highest infestation rate of ectoparasites in young animals due to poor grooming behaviour.

The SSG area has higher infestation rates than the NWC area, where more suitable conditions (temperature, pH, CO2, and moisture) exist for intermediate hosts. Additionally, female sheep and goats harboured more infestations than males; this variation could be because females stay longer in a flock or herd for reproduction and breeding. Our results were consistent with those of Shibeshi et al.^33^, who reported a higher prevalence of ectoparasites in females compared to males. In the present study, *C. felis* and *C. canis *were nearly prevalent in the two survey localities, where *C. felis* was the most common in the NWC area and *C. canis* was common in the SSG area, where cats and dogs were widespread, respectively, in the absence of rat fleas. *C. felis* was the most frequent flea species identified (98.8%) in Western Australia^35^ and in rural areas of South Africa^36^. *Ctenocephalides* sp. typically infests dogs, cats, and rodents, while goats and sheep are considered incidental hosts. Infestation may be detected during a sampling stemming from the presence of fleas from cats and dogs in the farm vicinity at the time of sampling^37^.

The purpose of this part of the study was to molecularly describe these pathogens using samples from all sexes and flea species to determine the genotypes of viruses in fleas parasitizing small animals in the study locations. When investigating vector-borne diseases with molecular methods, retention of the entire flea exoskeleton for taxonomic purposes is crucial since it provides readily available voucher specimens^38,39^. In the present investigation, the 70% alcohol renewal and crushing techniques that were used before PCR were suitable for studies concentrating on significant flea-transmitted pathogens and flea microbiomes.

Arthropods might spread SARS-CoV-2, according to researchers’ theories. The COVID-19 virus originated in bats, developed in pangolins, and then spread to humans^40^. No research has shown that blood-sucking arthropods such as mosquitoes can transmit SARS-CoV-2^41^, but tainted human waste or items used for personal care could be promising sources of viral transmission by flies and cockroaches^42,43^. To date, there has been no evidence that insects handle this type of transmission. Therefore, unless proven otherwise, they may serve as virus vectors, even though the data showed that PCR amplification had not occurred or had not created bands at the predicted level. The present findings supported the hypothesis that *Ctenocephalides* play an adverse role in the spread of coronaviruses*.*

Lumpy skin disease virus (LSDV) is a nonzoonotic and vector-borne disease. It affects and causes a considerable economic burden on cattle and buffaloes^4,44,45^. LSDV spreads more quickly during hot weather because vector transmission may vary between afflicted regions, and the principal mechanical vectors for the development of the virus are blood-sucking insects and ticks that frequently feed on cattle^46^. In contrast to prior studies in which LSDV was detected in goats in various areas of Egypt under accession numbers (MK342935, MN792930) by Mikhael and Ali^47^ and Zanaty et al.^48^ (GenBank database- https://www.ncbi.nlm.nih.gov/nucleotide/), LSDV was absent in all submitted isolates from different sexes and species of fleas in the present study.

For the detection of CaPV, two loop-mediated isothermal amplification (LAMP) methods have been used^49^. Such assays offer potential for diagnostic laboratories with few resources, even in the field^24^. More assays, including easy-to-use molecular testing techniques and PCR procedures appropriate for portable thermocyclers^50,51^, are expected to become accessible soon for epidemiological field research^52^. The GPCR gene can be sequenced as an alternative method for identifying field and vaccine viruses^2^.

In the present study, four reported isolates of the two *Ctenocephalides* species of all sexes produced four bands at 570 bp after PCR amplification using oligonucleotide primer sequences targeting the ORF103 gene of *Capripoxvirus*. Phylogenetic analysis of sheeppox virus revealed that five isolates from sheep collected in China and India^53^, Saudi Arabia, Iraq, and Nigeria^54^, and Egypt^55,56^ and maintained in the GenBank database were 100% identical. The GPV isolate has been genotyped, described, and confirmed to be 100% inappreciable to a different Egyptian reference strain recovered from goats and registered in GenBank under accession number MW546999 by Mikhael and Ali^47^. Our isolate also shares a close relationship with AY077836 from goats in Kazakhstan^57^, MN072622 from Turkey^54^, and KX576657 from a lamb in Iran^58^.

## Conclusion

The present study morphologically identified the subspecies and sexes of flea-infested small ruminants under a microscope and molecularly determined the genetic diversity and variability within and between different viruses. This study provides further insight into the versatility of Capripox viruses in adapting to various environmental conditions by detecting and identifying them in flea-infested sheep and goats on the Northern West Coast of Egypt and the South Sinai Governorate.

## Data Availability

Data will be available when requested from the corresponding author.

## Abbreviations

CaPVs: *Capripoxvirus*
GTPV: Goatpox virus
SPPV: Sheeppox virus
LSDV: Lumpy skin disease virus
BTV: Bluetongue
OIE: The World Organization for Animal Health
SARS-CoV-2: Severe Acute Respiratory Syndrome Coronavirus 2
